# Long-Term Imaging Follow-Up in DIPNECH: Multicenter Experience

**DOI:** 10.3390/jcm10132950

**Published:** 2021-06-30

**Authors:** Cécile Chung, Sébastien Bommart, Sylvain Marchand-Adam, Mathieu Lederlin, Ludovic Fournel, Marie-Christine Charpentier, Lionel Groussin, Marie Wislez, Marie-Pierre Revel, Guillaume Chassagnon

**Affiliations:** 1 Department of Radiology, AP-HP. Centre, Hôpital Cochin, 75014 Paris, France; cecile.chung1@gmail.com (C.C.); marie-pierre.revel@aphp.fr (M.-P.R.); 2 Université de Paris, 85 Boulevard Saint-Germain, 75006 Paris, France; ludovic.fournel@aphp.fr (L.F.); lionel.groussin@aphp.fr (L.G.); marie.wislez@aphp.fr (M.W.); 3 Radiology Department, CHU Montpellier, Hôpital Arnaud de Villeneuve, 34090 Montpellier, France; s-bommart@chu-montpellier.fr; 4 Université de Montpellier, PHYMEDEXP-INSERM U1046-CNRS UMR 9214, 34000 Montpellier, France; 5 Pulmonology Department, Université François Rabelais, CHU Tours, Hôpital Bretonneau, 37000 Tours, France; s.marchandadam@univ-tours.fr; 6 Department of Radiology, University of Rennes, University Hospital of Rennes, 35033 Rennes, France; mathieu.lederlin@chu-rennes.fr; 7 Thoracic Surgery Department, AP-HP. Centre, Hôpital Cochin, 75014 Paris, France; 8 Department of Pathology, AP-HP. Centre, Hôpital Cochin, 75014 Paris, France; marie-christine.charpentier@aphp.fr; 9 Department of Endocrinology, AP-HP. Centre, Hôpital Cochin, 75014 Paris, France; 10 Oncology Thoracic Unit Pulmonology Department, AP-HP. Centre, Hôpital Cochin, 75014 Paris, France; 11 Université de Paris, Centre de Recherche des Cordeliers, Inserm, «Inflammation, Complement, and Cancer», 75006 Paris, France

**Keywords:** carcinoid tumor, neoplasm metastasis, lymphatic metastasis, multidetector computed tomography, neuroendocrine cells

## Abstract

Diffuse pulmonary neuroendocrine cell hyperplasia (DIPNECH) is a rare pre-invasive disease whose pathophysiology remains unclear. We aimed to assess long-term evolution in imaging of DIPNECH, in order to propose follow-up recommendations. Patients with histologically confirmed DIPNECH from four centers, evaluated between 2001 and 2020, were enrolled if they had at least two available chest computed tomography (CT) exams performed at least 24 months apart. CT exams were analyzed for the presence and the evolution of DIPNECH-related CT findings. Twenty-seven patients, mostly of female gender (*n* = 25/27; 93%) were included. Longitudinal follow-up over a median 63-month duration (IQR: 31–80 months) demonstrated an increase in the size of lung nodules in 19 patients (19/27, 70%) and the occurrence of metastatic spread in three patients (3/27, 11%). The metastatic spread was limited to mediastinal lymph nodes in one patient, whereas the other two patients had both lymph node and distant metastases. The mean time interval between baseline CT scan and metastatic spread was 70 months (14, 74 and 123 months). Therefore, long-term annual imaging follow-up of DIPNECH might be appropriate to encompass the heterogeneous longitudinal behavior of this disease.

## 1. Introduction

Diffuse pulmonary neuroendocrine hyperplasia (DIPNECH) is presumed to be a rare pulmonary disorder characterized by a diffuse proliferation of pulmonary neuroendocrine cells of the airway mucosa. The proliferation may be limited to the mucosa or locally cross the basement membrane to form tumorlets or develop into carcinoid tumors [1]. Additionally, DIPNECH may result in small airway obstruction due to the combination of luminal protrusion of the neuroendocrine cells and constrictive bronchiolitis related to peribronchiolar fibrosis induced by the secretion of amines and peptides [2]. DIPNECH has been recognized as a separate entity in 1992 [3] and is distinct from reactive pulmonary neuroendocrine cells hyperplasia that can be observed in several conditions, including chronic lung disease, cigarette smoking and living at high altitude [3]. In a series of 1090 patients who received resection for primary lung carcinomas, Ruffini et al. found DIPNECH in 3 patients out of 55 with lung carcinoid tumor having a prevalence of 5.4% in this subgroup [4]. While being recognized as a pre-invasive condition for lung carcinoid tumors by the World Health Organization (WHO) [1] and being increasingly reported, DIPNECH pathophysiology remains poorly understood. 

The first uncertainty regarding this disease concerns its exact definition, and to date, there is no consensus on diagnostic criteria. The WHO definition [1] is strictly based on histopathology and encompasses patients with different clinical presentations. Indeed, about one-half of patients present with pulmonary symptoms and functional impairment, whereas the other half of patients remain asymptomatic, according to the literature [5,6]. At the same time, some asymptomatic patients may present the computed tomography (CT) abnormalities usually encountered in DIPNECH (e.g., mosaic perfusion, multiple nodules and micronodules), others only present neuroendocrine hyperplasia on histopathologic examinations, and no CT anomalies. Some authors have proposed to differentiate between i/ DIPNECH syndrome in case of symptomatic diffuse pulmonary neuroendocrine cell proliferation on histology, ii/ DIPNECH in case of asymptomatic diffuse pulmonary neuroendocrine cell proliferation on histology with suggestive radiological features, and iii/ pulmonary neuroendocrine cell hyperplasia (PNECH) in case of asymptomatic diffuse pulmonary neuroendocrine cell proliferation on histology without compatible radiological findings [7]. The lack of symptoms in DIPNECH patients with signs of small airway disease on chest CT is related to the fact that small airways represent the “silent zone” of the lung where the disease might accumulate for many years with very little effect [8,9]. Chest CT and functional magnetic resonance imaging are reported to have better sensitivity than spirometry for early detection of small airway disease, as reported in cystic fibrosis children [10,11].

Uncertainties regarding DIPNECH also include the pathophysiological mechanisms and the appropriate treatment as well as long-term evolution and risk factors for poor outcome. Although DIPNECH is generally considered to be an indolent disease, some patients develop respiratory failure leading to lung transplantation [6,12] or metastatic spread [13]. Given the scarcity of longitudinal data available, there are currently no specific guidelines for DIPNECH imaging follow-up.

Therefore, the aim of this study was to assess long-term evolution in imaging in a cohort of patients with histologically proven DIPNECH, which could serve as a basis for follow-up recommendations.

## 2. Materials and Methods

### 2.1. Study Population

This retrospective multicenter study was approved by our Institutional Review Board (AAA-2019-08012) which waived the need for patients’ consent.

Clinical, pathological and radiological records from 4 University hospitals were reviewed for patients with a histologically confirmed diagnosis of DIPNECH over a 19-year period, from 2001 to 2020. The diagnosis of DIPNECH encompassed DIPNECH and DIPNECH syndrome and required at least one of the following two criteria:

- either diffuse pulmonary neuroendocrine cells hyperplasia on a surgical biopsy or lung resection specimen;
- or presence of multiple pulmonary nodules on CT with one proven carcinoid lung tumor;

and:

- either symptomatic chronic obstructive airway disease in the absence of other etiology;
- or diffuse mosaic perfusion on CT in the absence of other etiology.

Exclusion criteria were the unavailability of two chest CT scans performed at least 24 months apart and the association with an unrelated active cancer.

### 2.2. Image Analysis

The baseline and the latest chest CT exams were retrospectively analyzed. In the case of surgical resection between these 2 CT examinations, the CT exam performed just before surgery was also analyzed. All CT exams were read in consensus by a junior radiologist (CC) and a chest radiologist (GC) with 1 and 7-year experience in chest imaging, respectively. 

Baseline CT exams were analyzed for the presence of the seven different CT features reported in DIPNECH [14], namely: mosaic perfusion, pulmonary nodules, subpleural atelectasis, mucoid impactions, bronchial thickening, bronchiectasis and pulmonary cysts. Regarding the pulmonary nodules, micronodules/nodules suggestive of tumorlets (<5 mm in diameter) and nodules suggestive of carcinoid tumors (≥5 mm in diameter) were counted, the size of the largest nodule was measured and the presence of calcifications, as well as central/peripheral location, were assessed. A nodule was considered to be centrally located if it was connected to a segmental or more proximal bronchus. Side-by-side comparison between baseline and follow-up CT exams was also performed to determine changes in mosaic perfusion extent, changes in size or number of pulmonary nodules and occurrence of lymphatic or metastatic spread.

### 2.3. Clinical Data

Patient charts were reviewed for demographic characteristics, symptoms, smoking history and pulmonary function tests (PFTs) at the time of the baseline CT scan. The diagnosis circumstances were investigated and classified into 3 categories: incidental finding, respiratory symptom or CT staging of unrelated neoplasm. The date and type of surgery, as well as histopathological results, were also retrieved. 

In cases where another surgery was performed after the diagnosis of DIPNECH had already been established, the CT examination performed prior to treatment and clinical information were both reviewed.

Additionally, the latest follow-up PFTs were collected to assess the evolution of obstruction based on forced expiratory volume in 1 second (FEV_1_). A decrease of at least 10% of FEV_1_ absolute predicted value was considered significant.

### 2.4. Statistical Analysis

Statistical analysis was performed using R software (version 3.6, R Foundation, Vienna, Austria). Qualitative variables were expressed as percentages, whereas quantitative variables were expressed as the median and interquartile range (IQR). Patient characteristics and baseline CT findings were compared between patients with progressive disease requiring additional intervention and the remaining patients. Comparisons were performed using the Fisher exact test for qualitative variables and the Wilcoxon–Mann–Whitney test for quantitative variables. *p* values less than 0.05 were regarded as being significant.

## 3. Results

### 3.1. Patients’ Characteristics at Baseline

**Figure 1:**
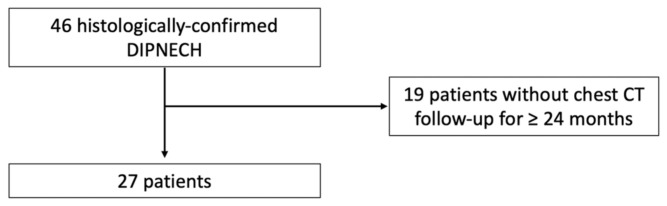
Flow chart.

A total of 66 patients considered to have DIPNECH were identified in the databases of four university hospitals. Of these, the 27 patients who had at least two years of CT follow-up and met the criteria for DIPNECH diagnosis were included (Figure 1, flow chart). Patients were mostly of female gender (*n* = 25/27; 93%) and had a median age of 63 years (IQR: 59–72 years; range: 42–88 years) at the time of baseline CT scan (Table 1).

Neuroendocrine proliferation was histologically confirmed on surgical resection specimen in 25 patients (93%), CT-guided transthoracic biopsy in one patient (4%) and ultrasound-guided biopsy of liver metastasis in one patient (4%). All patients in this series had at least one histologically confirmed lung carcinoid tumor. Atypical carcinoids were observed in six patients (*n* = 6/27; 22%), whereas only typical carcinoids were found in the remaining 21 patients with the limit that not all nodules were resected.

For the 24 patients for whom the information about circumstances of diagnosis was available, the majority were diagnosed because of thoracic symptoms (*n* = 15/24; 62%), whereas for the other nine patients, nodules were found at the initial staging of an unrelated neoplasm (*n* = 5/24; 21%) or incidentally (*n* = 4/24; 17%).

The majority of patients for whom the information was available were symptomatic (*n* = 18/22; 82%) with cough, dyspnea or other respiratory symptoms in 12 (*n* = 12/22; 55%), 10 (*n* = 10/22; 45%) and 8 patients (*n* = 8/22; 36%), respectively. Other respiratory symptoms consisted in recurrent bronchitis (*n* = 3/8; 38%) and unrelated obstructive sleep apnea (*n* = 3/8; 38%). The median delay from symptoms onset to the first CT scan was 56 months (IQR: 27–90 months; range: 0–274 months).

Most patients were non-smokers (*n* = 11/20; 55%). Baseline PFTs were available for 18 patients, showing an obstructive syndrome in 9 of them (*n* = 9/18; 50%). The median FEV_1_ was 82 percent of the predicted value (IQR: 72–89%; range: 26–110%).

### 3.2. Imaging Findings at Baseline

All but two available baseline CT scans (25/27; 93%) were performed prior to any surgery. All patients had pulmonary nodules associated with mosaic perfusion (*n* = 27/27, 100%) (Table 2). 

A total of 20 patients (*n* = 20/27, 74%) had 10 or more nodules measuring less than 5 mm, which are conventionally considered tumorlets, while 3 patients (*n* = 3/27, 11%) had 10 or more nodules measuring at least 5 mm, which are conventionally considered as carcinoid tumors. The remaining 7 patients had less than 10 nodules, with a median number of tumorlets and carcinoid tumors of 3 (IQR = 1–3, range: 0–7) and 2 (IQR= 1–4, range: 0–8), respectively. Only three patients had three or fewer lung nodules (tumorlets + carcinoids) on baseline chest CT, but these patients also had diffuse mosaic perfusion related to the diffuse neuroendocrine cell hyperplasia. In all three cases, the diagnosis has been confirmed by surgical biopsy showing in addition to the carcinoid tumors, multiple tumorlets (some being beyond CT resolution) and diffuse neuroendocrine cell hyperplasia. The median size of the largest nodule was 9 mm (IQR = 8–13 mm, range: 2–32 mm). Centrally located nodules, suggesting central carcinoid tumors, and calcifications were found in only three patients (*n* = 3/27, 11%).

Other features of airway involvement included band-like subpleural atelectasis in 67% of patients (*n* = 18/27), mucoid impaction in 37% (*n* = 10/27), mild bronchial thickening in 33% (*n* = 9/27) and mild cylindrical bronchiectasis in 19% (*n* = 5/27). In one patient (*n* = 1/27, 4%), the chest CT scan revealed the presence of pulmonary cysts, but pathologic evaluation of these cysts was not available. Baseline CT exams revealed no evidence of lymphatic or extrapulmonary dissemination.

### 3.3. Disease Evolution and Treatment

The median duration of radiological follow-up was 63 months (IQR: 31–80 months; range: 24–170 months). During this period, lung nodules increased in 19 patients (*n* = 19/27; 70%) of whom 17 (17/27, 63%) presented an increased number and 18 (18/27, 67%) a size increase, whereas nodules remained stable in the remaining eight patients (*n* = 8/27; 30%). Despite nodule increase in the majority of patients, only two patients had an additional local treatment. In the first patient, DIPNECH had been diagnosed 15 years (176 months) earlier on a surgical biopsy. The patient had Cushing’s syndrome and a stable 18 mm lung nodule in the right lower lobe, which was percutaneously biopsied. The pathological study demonstrated an atypical lung carcinoid showing ACTH antibody staining and the patient was treated by stereotactic body radiotherapy. Following treatment, Cushing’s disappeared but relapsed six months later. In the second patient, DIPNECH had been diagnosed 62 months earlier after wedge resections in the right upper, middle and lower lobes showed three atypical lung carcinoids along with tumorlets and pulmonary neuroendocrine cell hyperplasia. The patient was re-operated on 25 months later, for the resection of a newly appeared 15 mm nodule, which turned out to be a granuloma.

With regard to CT signs of small airway involvement, mosaic perfusion and bronchial dilatations remained stable in the majority of patients (*n* = 22/27; 81% and *n* = 26/27; 96%, respectively).

Lymph node enlargement suggestive of lymphatic spread was observed and confirmed in three patients (*n* = 3/27, 11%). All had at least one atypical lung carcinoid (*n* = 3/3; 100%).

**Figure 2:**
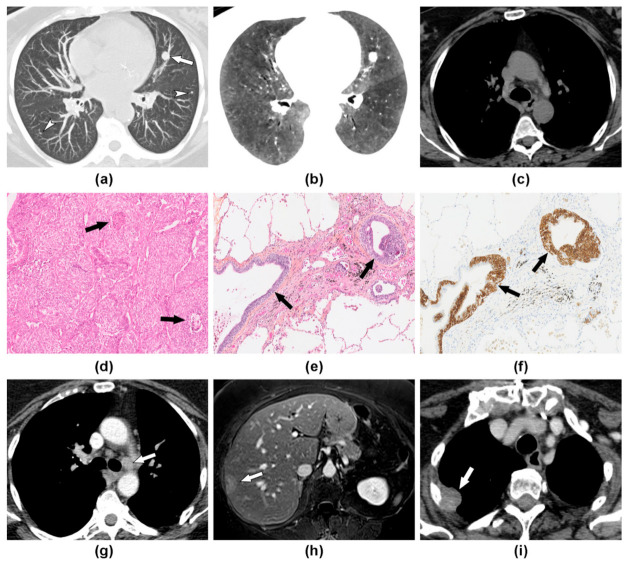
DIPNECH with metastatic course over an 8 year period in a 60-year-old woman. (**a**) Maximum intensity projection (MIP) reformation of baseline chest CT scan shows several nodules (arrow and arrowheads), including a 12 mm nodule in the left upper lobe (arrow). (**b**) Minimum intensity projection (MinIP) reformation reveals the characteristic association to diffuse mosaic perfusion. (**c**) There was no mediastinal lymph node enlargement. (**d**) Histopathological analysis of the 12 mm nodule shows carcinoid tumor with necrosis spots (arrows) consistent with an atypical form. (**e**,**f**) Analysis of distant parenchyma revealed bronchial cell proliferation (arrows) on hematoxylin and eosin stain (E), which corresponded to neuroendocrine cells hyperplasia on chromogranin A stain (F). (**g**) Five years later, contrast-enhanced CT shows the development of mediastinal lymphadenopathy leading to the new surgery. (**h**) Two years later, Gadolinium-enhanced T1 weighted axial image demonstrates liver metastasis leading to the start of chemotherapy. (**i**) Contrast-enhanced CT scan performed 6 months later shows disease progression with the occurrence of pleural metastasis (arrow). Nodal and liver metastasis were histologically confirmed and showed features of atypical lung carcinoid.

Distant metastases occurred in two patients (*n* = 2/27, 7%), all of whom also had lymphatic dissemination. Distant and lymphatic metastases were synchronous in one patient, while in another patient, the nodal spread had preceded distant metastases by 25 months (Figure 2). Both patients developed liver metastasis and then pleural carcinomatosis. In one patient, the lung carcinoid was also metastatic to the bone. 

Pathological confirmation of the metastatic stage was obtained for all patients and showed features of atypical carcinoid for all.

Therefore, three patients (*n* = 3/27, 11%) developed lymphatic spread with or without distant metastases, in a mean time interval time of 67 months following the baseline CT scan (14, 63 and 123 months). The frequency of histologically confirmed atypical carcinoids was significantly higher in patients who developed lymphatic or distant metastases (100% vs. 13%; *p* = 0.005). The other patient characteristics were not significantly different when comparing these two patient categories (*p* > 0.05) (Table 1 and Table 2).

Seven patients had baseline and follow-up PFTs with a median follow-up interval of 51 months (IQR: 38–79 months; range: 24–97 months). Functional worsening was observed in four patients (*n* = 4/7; 57%) with a median FEV_1_ decrease of −14.5% in absolute predicted value (−35%, −15%, −14% and −13%), whereas FEV_1_ remained stable or improved in the remaining three patients (−4%, −3% and +13% in absolute predicted value).

## 4. Discussion

In this multicenter study, we found that DIPNECH was a progressive disease in the majority of patients, over a median follow-up time of 63 months, with an increase in the size and/or number of lung nodules. Despite this evolution, the disease course remained indolent in all but three patients (11%) who developed lymphatic spread, followed by a distant metastatic spread in two patients.

To the best of our knowledge, this multicenter study is the first to focus on long-term CT follow-up in DIPNECH, and our cohort is one of the largest reported to date [5,6,15,16,17,18,19,20]. As in other reported series, we found a strong female predominance with 93% of women in our cohort compared to 79 to 100% in the literature [6,15]. Most patients (82%) were symptomatic (DIPNECH syndrome) and all had mosaic perfusion on chest CT scan, a sign of small airway disease. The median 56-month delay between the onset of symptoms and DIPNECH diagnosis is in phase with previous reports. Indeed, many patients are misdiagnosed as having late-onset asthma. However, the association of multiple lung nodules and mosaic perfusion observed in all our patients should be considered as highly suggestive of DIPNECH in middle-aged women. This association is part of the diagnostic criteria proposed by Carr et al., allowing DIPNECH to be diagnosed without histopathological confirmation [15]. 

Unlike some other studies, mosaic perfusion was present in all patients of our series (100% vs. 17–100%) [2,15,21]. Mosaic perfusion, which is due to vasoconstriction in areas of small airway obstruction and blood flow redistribution to normal areas [22], may be subtle on native CT images and standard lung windowing. Its detection is enhanced by the use of minimum intensity projection (minIP) reformation with narrowed window width and optimized center level settings [14,23].

Disease evolution in DIPNECH may be related to either small airway disease worsening or malignant progression. Several studies have reported an increase of pulmonary symptoms or a decline in pulmonary function in 7 to 55% of patients [5,6,15]; for some patients, functional worsening leads to end-stage respiratory failure, lung transplantation or death. Gorshtein et al. reported that 55% of patients had both functionally and radiologically progressive disease, the other remaining stable [5]. We observed increased mosaic perfusion in 19% of our patients and functional worsening in 57% of patients who had PFTs re-evaluation. By contrast, the majority of patients presented an increase in pulmonary nodules. Only two series have reported imaging follow-up data in DIPNECH patients and found a progression of lung lesions in 49 and 55% of cases but over shorter median follow durations (19.5 and 56 months vs. 63 months) [5,24]. While none of our patients were metastatic at the time of diagnosis, 11% developed a metastatic nodal or extranodal spread during the disease course. Only a few cases of metastatic spread have been reported to date in DIPNECH, the prevalence widely ranging from 0 to 27% [5,17]. The reported cases mostly consist of lymphatic spread, with only three previously reported cases of extranodal metastasis in the eye and bone [17], the adrenal gland [5] and the liver [13]. Similar to previous reports, we found metastatic disease to occur in patients with proven atypical carcinoid tumors, which was the only characteristic significantly associated with a metastatic evolution. The presence of atypical carcinoid is reported to be rare in DIPNECH, with a prevalence ranging from 0 to 27% [5,6,19]. However, in the setting of multiple bilateral nodules, not every nodule can be pathologically evaluated, and thus, the proportion of patients with atypical histology may be underestimated. Similarly, it is not possible to exclude that some typical carcinoids could metastasize, since lymphatic spread has been reported in 5% of resected typical lung carcinoid [25].

**Figure 3:**
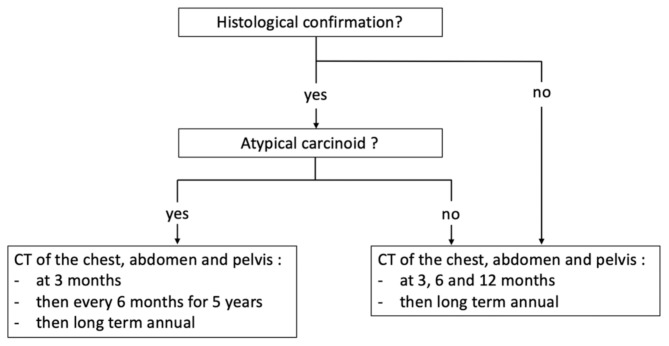
Proposal of follow-up recommendations for patients with DIPNECH.

In view of the long delay, up to 123 months, between baseline CT and the occurrence of metastatic spread, long-term CT surveillance should be performed in DIPNECH. According to the current recommendations of the European Neuroendocrine Tumor Society (ENETS) on pulmonary carcinoids [26], CT scan should be performed at 3 and 6 months after treatment and then every 12 months for the first 2 years in the setting of typical pulmonary carcinoid. Then long-term annual chest X-ray and CT every 3 years are recommended. For atypical pulmonary carcinoids, closer monitoring is recommended with CT imaging 3 months post-surgery and then every 6 months for 5 years. After 5 years, an annual CT should be performed. In the setting of DIPNECH, atypical pulmonary carcinoids are presumed to be rare, and due to the diffuse nature of the disease and the advanced patient age, resection of all lesions is usually not feasible. Furthermore, an increasing number of patients are being diagnosed with DIPNECH without surgical resection, potentially leaving in place atypical carcinoids. Therefore, close long-term monitoring seems necessary in these patients. Based on our experience and previous reports, we propose contrast-enhanced CT of the chest, abdomen and pelvis at 3 months, 6 months and 12 months after baseline CT, and then long-term annual CT (Figure 3). In the case of confirmed atypical pulmonary carcinoid, the ENETS follow-up recommendations should be applied.

Our study has several limitations; first, because of its retrospective design and the lack of available recommendations for DIPNECH follow-up, there was no standardization of imaging follow-up, and several patients could not be included due to the lack of 2-year follow-up. Others were not included because histological confirmation was lacking. We chose to propose recommendations based only on histologically proven follow-up cases. Additionally, the contribution of the 68Ga DOTATOC PET-CT scanner could not be evaluated because only a few patients underwent PET/CT in this multicenter cohort study. Finally, the limited size of our cohort made it impossible to identify significant differences other than the presence of atypical carcinoid between patients with and without metastatic spread.

## 5. Conclusions

In conclusion, the majority of DIPNECH patients present long-term disease progression on imaging and 11% develop metastatic spread at 63-month median follow-up. Long-term imaging follow-up is therefore required. Further prospective studies are needed to evaluate these follow-up recommendations. 

## Figures and Tables

**Table 1:** Patient characteristics.

	All (*n* = 27)	Lymphatic or Distant Metastasis (*n* = 3)	No Lymphatic or Distant Metastasis (*n* = 24)	*p* Value *
Female	25/27 (93)	3/3 (100%)	22/24 (92%)	1
Age * (years)	63 (59–72)	62 (61–67)	64 (59–72)	0.969
≥1 atypical carcinoid	6/27 (22%)	3/3 (100%)	3/24 (13%)	0.005
** Circumstances of the Diagnosis: **				0.162
Symptomatic patient	15/24 (62%)	1/3 (33%)	14/21 (67%)	
Incidental finding	5/24 (21%)	1/3 (33%)	4/21 (19%)	
Staging of unrelated neoplasm	4/24 (17%)	1/3 (33%)	3/21 (14%)	
**Respiratory Symptoms:**	18/22 (82%)	2/3 (67%)	16/19 (84%)	0.470
Cough	12/22 (55%)	1/3 (33%)	11/19 (58%)	0.571
Dyspnea	10/22 (45%)	0/3 (0%)	10/19 (53%)	0.221
Other respiratory symptoms	8/22 (36%)	1/3 (33%)	7/19 (37%)	1
Non-smoker	11/20 (55%)	2/2 (100%)	9/18 (50%)	0.479
**Baseline PFT:**				
Obstructive syndrome	9/18 (50%)	2/2 (100%)	7/16 (44%)	0.471
FEV_1_ (% pred)	82 (72–89)	79 (78–80)	84 (63–90)	0.574

Note: Quantitative data are presented as median [interquartile range] and qualitative data are presented as proportion (percentage). * at time of the baseline computed tomography scan. FEV1: forced expiratory volume in 1 second, PFT: pulmonary function test.

**Table 2:** Imaging findings in DIPNECH patients.

	All (*n* = 27)	Lymphatic or Distant Metastasis (*n* = 3)	No Lymphatic or Distant Metastasis (*n* = 24)	*p* Value *
**Baseline CT Scan**				
Mosaic perfusion	27/27 (100%)	3/3 (100%)	24/24 (100%)	1
Pulmonary nodules	27/27 (100%)	3/3 (100%)	24/24 (100%)	1
Pulmonary nodules < 5 mm	26/27 (96%)	3/3 (100%)	23/24 (96%)	1
≥10 nodules	20/27 (74%)	2/3 (67%)	18/24 (75%)	1
Pulmonary nodules ≥ 5 mm	24/27 (89%)	3/3 (100%)	21/24 (88%)	1
≥10 nodules	3/27 (11%)	0/3 (0%)	3/24 (13%)	1
Size of the largest nodule, mm	9 (8–13)	10 (9–11)	9 (8–17)	0.611
≥1 centrally located nodule	3/27 (11%)	0/3 (0%)	3/24 (13%)	1
≥1 calcified nodule	3/27 (11%)	0/3 (0%)	3/24 (13%)	1
Subpleural atelectasis	18/27 (67%)	2/3 (67%)	16/24 (67%)	1
Mucoid impaction	10/27 (37%)	1/3 (33%)	9/24 (38%)	1
Bronchial thickening	9/27 (33%)	1/3 (33%)	8/24 (33%)	1
Bronchiectasis	5/27 (19%)	1/3 (33%)	4/24 (17%)	0.474
Pulmonary cysts	1/27 (4%)	0/3 (0%)	1/24 (4%)	1
Lymph node enlargement	0/27 (0%)	0/3 (0%)	0/24 (0%)	1
Distant metastasis	0/27 (0%)	0/3 (0%)	0/24 (0%)	1
Interval time between baseline and follow-up chest CT (months)	63 (31–80)	101 (62–110)	62 (32–78)	0.563
**Disease Evolution on CHESt CT**				
Increase in mosaic perfusion	5/27 (19%)	0/3 (0%)	5/24 (21%)	1
Increase in nodule size	18/27 (67%)	3/3 (100%)	15/24 (63%)	0.529
Increase in number of nodules	17/27 (63%)	3/3 (100%)	14/24 (58%)	0.274
Lymph node enlargement	3/27 (11%)	3/3 (100%)	0/24 (0%)	<0.001
Distant metastasis	2/27 (7%)	2/3 (67%)	0/24 (0%)	0.009

Note: Quantitative data are presented as median (interquartile range) and qualitative data are presented as proportion (percentage). * at time of the baseline computed tomography scan. CT = computed tomography.

## Data Availability

Not applicable.

## References

[1] Travis W.D., International Agency for Research on Cancer (2015). WHO Classification of Tumours of Lung, Pleura, Thymus and Heart.

[2] Nassar A.A., Jaroszewski D.E., Helmers R.A., Colby T.V., Patel B.M., Mookadam F. (2011). Diffuse Idiopathic Pulmonary Neuroendocrine Cell Hyperplasia: A Systematic Overview. Am. J. Respir. Crit. Care Med..

[3] Aguayo S.M., Miller Y.E., Waldron J.A., Bogin R.M., Sunday M.E., Staton G.W., Beam W.R., King T.E. (1992). Brief Report: Idiopathic Diffuse Hyperplasia of Pulmonary Neuroendocrine Cells and Airways Disease. N. Engl. J. Med..

[4] Ruffini E., Bongiovanni M., Cavallo A., Filosso P.L., Giobbe R., Mancuso M., Molinatti M., Oliaro A. (2004). The Significance of Associated Pre-Invasive Lesions in Patients Resected for Primary Lung Neoplasms. Eur. J. Cardio Thorac. Surg. Off. J. Eur. Assoc. Cardio-Thorac. Surg..

[5] Gorshtein A., Gross D.J., Barak D., Strenov Y., Refaeli Y., Shimon I., Grozinsky-Glasberg S. (2012). Diffuse Idiopathic Pulmonary Neuroendocrine Cell Hyperplasia and the Associated Lung Neuroendocrine Tumors: Clinical Experience with a Rare Entity. Cancer.

[6] Davies S.J., Gosney J.R., Hansell D.M., Wells A.U., du Bois R.M., Burke M.M., Sheppard M.N., Nicholson A.G. (2007). Diffuse Idiopathic Pulmonary Neuroendocrine Cell Hyperplasia: An under-Recognised Spectrum of Disease. Thorax.

[7] Rossi G., Cavazza A., Spagnolo P., Sverzellati N., Longo L., Jukna A., Montanari G., Carbonelli C., Vincenzi G., Bogina G. (2016). Diffuse Idiopathic Pulmonary Neuroendocrine Cell Hyperplasia Syndrome. Eur. Respir. J..

[8] Hogg J.C. (2004). Pathophysiology of Airflow Limitation in Chronic Obstructive Pulmonary Disease. Lancet.

[9] Burgel P.-R. (2011). The Role of Small Airways in Obstructive Airway Diseases. Eur. Respir. Rev..

[10] Brody A.S. (2004). Early Morphologic Changes in the Lungs of Asymptomatic Infants and Young Children with Cystic Fibrosis. J. Pediatr..

[11] Mall M.A., Stahl M., Graeber S.Y., Sommerburg O., Kauczor H.-U., Wielpütz M.O. (2016). Early Detection and Sensitive Monitoring of CF Lung Disease: Prospects of Improved and Safer Imaging: Early Detection and Sensitive Monitoring of CF. Pediatr. Pulmonol..

[12] Walker C.M., Vummidi D., Benditt J.O., Godwin J.D., Pipavath S. (2012). What Is DIPNECH?. Clin. Imaging.

[13] Flint K., Ye C., Henry T.L. (2019). Diffuse Idiopathic Pulmonary Neuroendocrine Cell Hyperplasia (DIPNECH) with Liver Metastases. BMJ Case Rep..

[14] Chassagnon G., Favelle O., Marchand-Adam S., De Muret A., Revel M.P. (2015). DIPNECH: When to Suggest This Diagnosis on CT. Clin. Radiol..

[15] Carr L.L., Chung J.H., Achcar R.D., Lesic Z., Rho J.Y., Yagihashi K., Tate R.M., Swigris J.J., Kern J.A. (2015). The Clinical Course of Diffuse Idiopathic Pulmonary Neuroendocrine Cell Hyperplasia. Chest.

[16] Marchevsky A.M., Wirtschafter E., Walts A.E. (2015). The Spectrum of Changes in Adults with Multifocal Pulmonary Neuroendocrine Proliferations: What Is the Minimum Set of Pathologic Criteria to Diagnose DIPNECH?. Hum. Pathol..

[17] Aubry M.-C., Thomas C.F., Jett J.R., Swensen S.J., Myers J.L. (2007). Significance of Multiple Carcinoid Tumors and Tumorlets in Surgical Lung Specimens. Chest.

[18] Myint Z.W., McCormick J., Chauhan A., Behrens E., Anthony L.B. (2018). Management of Diffuse Idiopathic Pulmonary Neuroendocrine Cell Hyperplasia: Review and a Single Center Experience. Lung.

[19] Mengoli M.C., Rossi G., Cavazza A., Franco R., Marino F.Z., Migaldi M., Gnetti L., Silini E.M., Ampollini L., Tiseo M. (2018). Diffuse Idiopathic Pulmonary Neuroendocrine Cell Hyperplasia (DIPNECH) Syndrome and Carcinoid Tumors with/without NECH: A Clinicopathologic, Radiologic, and Immunomolecular Comparison Study. Am. J. Surg. Pathol..

[20] Trisolini R., Valentini I., Tinelli C., Ferrari M., Guiducci G.M., Parri S.N.F., Dalpiaz G., Cancellieri A. (2016). DIPNECH: Association between Histopathology and Clinical Presentation. Lung.

[21] Lee J.S., Brown K.K., Cool C., Lynch D.A. (2002). Diffuse Pulmonary Neuroendocrine Cell Hyperplasia: Radiologic and Clinical Features. J. Comput. Assist. Tomogr..

[22] Ryu J.H., Myers J.L., Swensen S.J. (2003). Bronchiolar Disorders. Am. J. Respir. Crit. Care Med..

[23] Abbott G.F., Rosado-de-Christenson M.L., Rossi S.E., Suster S. (2009). Imaging of Small Airways Disease. J. Thorac. Imaging.

[24] Almquist D.R., Sonbol M.B., Kosiorek H., Halfdanarson T., Ross H.J., Jaroszewski D. (2020). Clinical Characteristics of DIPNECH: A Retrospective Analysis. Chest.

[25] Cusumano G., Fournel L., Strano S., Damotte D., Charpentier M.C., Galia A., Terminella A., Nicolosi M., Regnard J.F., Alifano M. (2017). Surgical Resection for Pulmonary Carcinoid: Long-Term Results of Multicentric Study—The Importance of Pathological N Status, More than We Thought. Lung.

[26] Caplin M.E., Baudin E., Ferolla P., Filosso P., Garcia-Yuste M., Lim E., Oberg K., Pelosi G., Perren A., Rossi R.E. (2015). Pulmonary Neuroendocrine (Carcinoid) Tumors: European Neuroendocrine Tumor Society Expert Consensus and Recommendations for Best Practice for Typical and Atypical Pulmonary Carcinoids. Ann. Oncol..

